# Supplementary figures and images for: Combination treatment of dendrosomal nanocurcumin and low-level laser therapy develops proliferation and migration of mouse embryonic fibroblasts and alter TGF-β, VEGF, TNF-α and IL-6 expressions involved in wound healing process
Source: PLoS One. 2021 May 6;16(5):e0247098. doi: 10.1371/journal.pone.0247098 (PMC8101758; doi:10.1371/journal.pone.0247098)

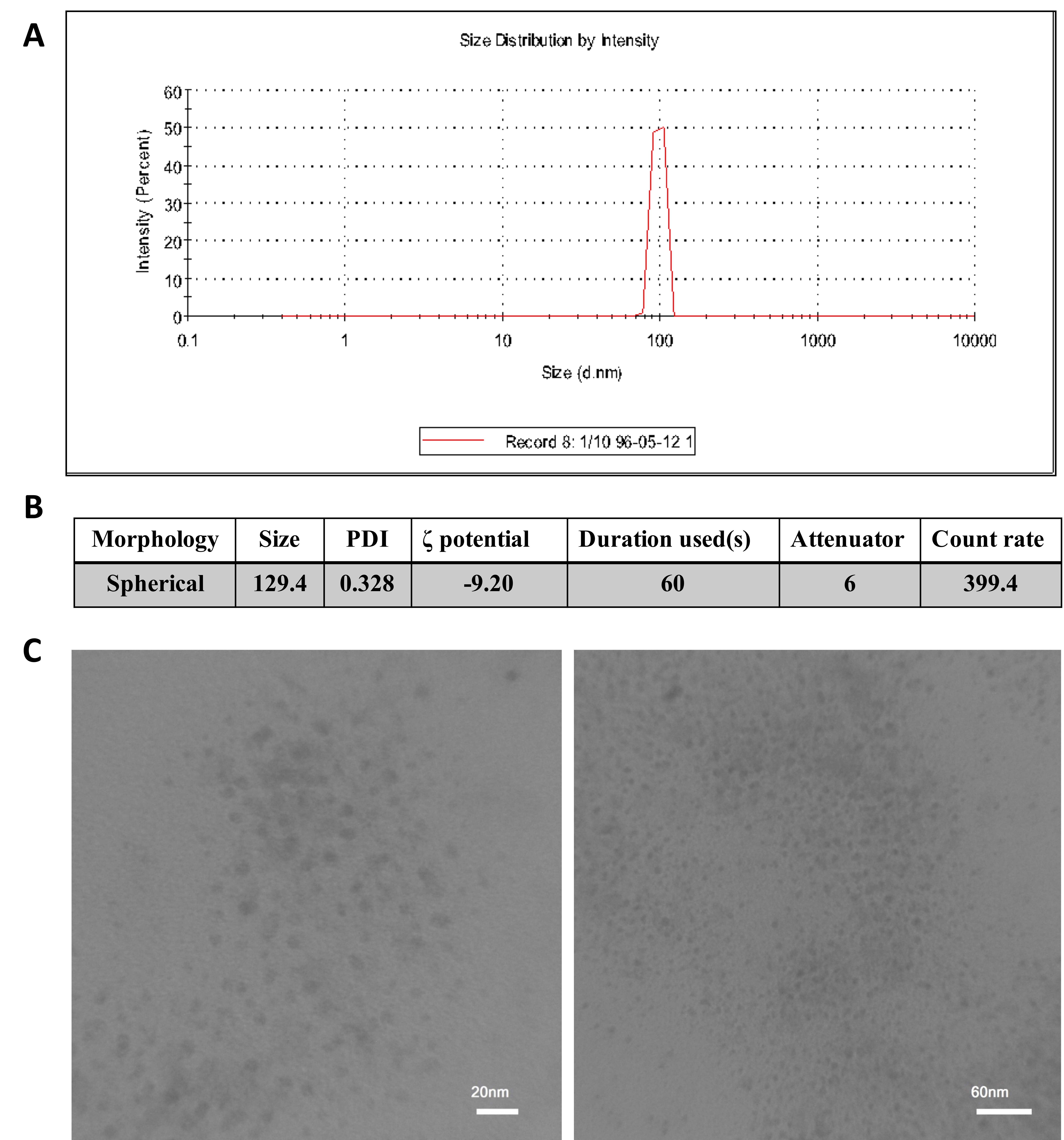

Supplement: S1 Fig — (A): DLS diagram of dendrosomal nanocurcumin (DNC) in which was analyzed in terms of size distribution by intensity of light scattering. With regard to DLS measurements (hydrodynamic radius), dendrosome particles size are majorly around 100 nm. (B): The size, ζ-potential and polydispersity index (PDI) of DNC. (C): Transmission electron micrographs (TEM) of DNC: spherical shape of DNC which indicate the compact form of micelles-carriers. (JPG) [file pone.0247098.s002.jpg]

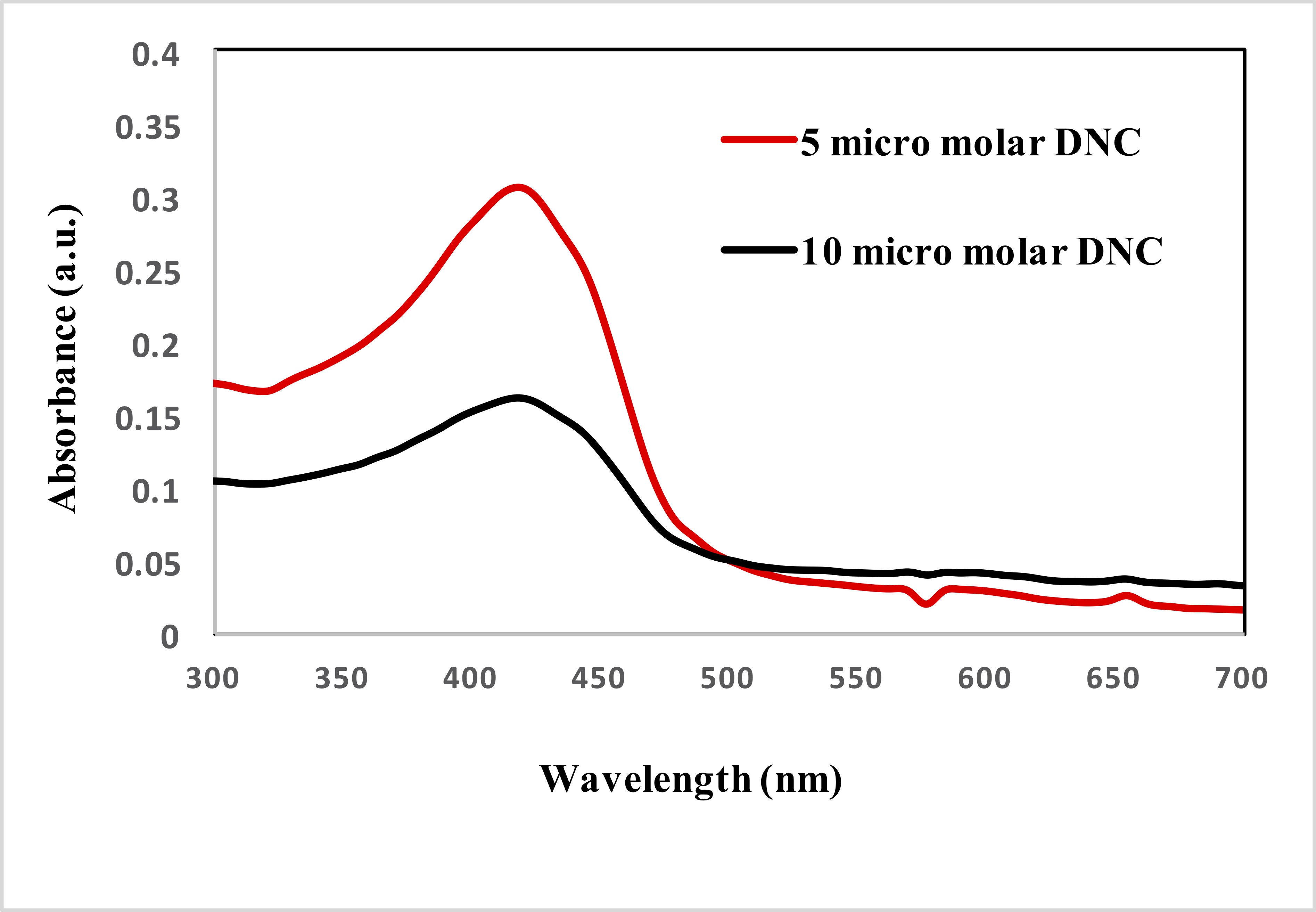

Supplement: S2 Fig — The UV–Vis spectra of Dendrosomal Nano Curcumin (DNC) dissolved in PBS with concentrations of 5 and 10 μM (anti-oxidant doses) represents that its light absorption spectrum is a broad band (300–500 nm) with maximum absorbance peak at a wavelength ~425 nm. Also, the more the DNC solution is diluted, the more the UV–VIS absorption intensity decreases. However, the wavelength of light absorption remains constant. (JPG) [file pone.0247098.s003.jpg]

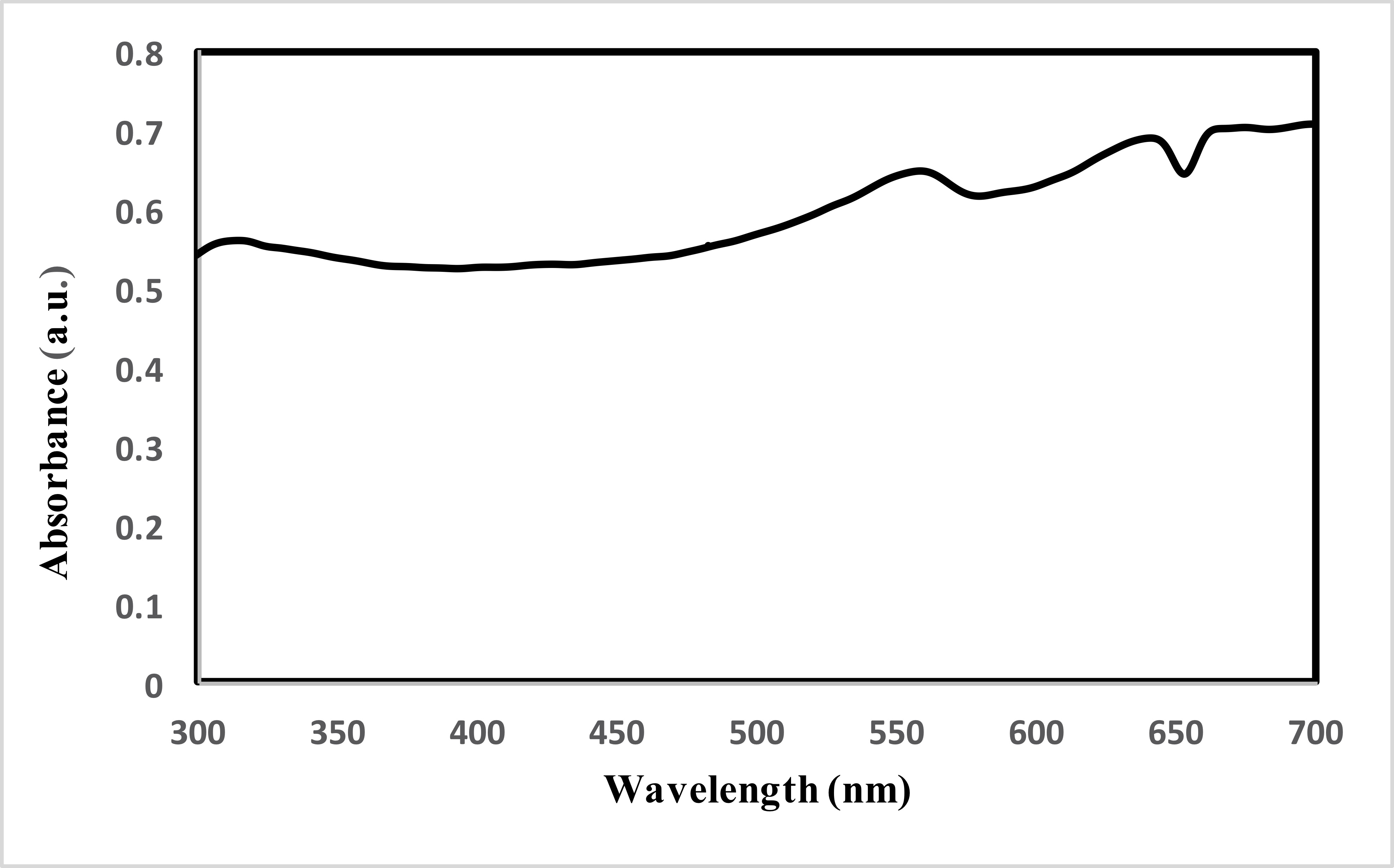

Supplement: S3 Fig — Absorbance measurement by UV–VIS for MEFs suspended in cell culture medium (DMEM) shows that absorption spectrum of MEFs is uniformly a broad band above 300 nm. (JPG) [file pone.0247098.s004.jpg]

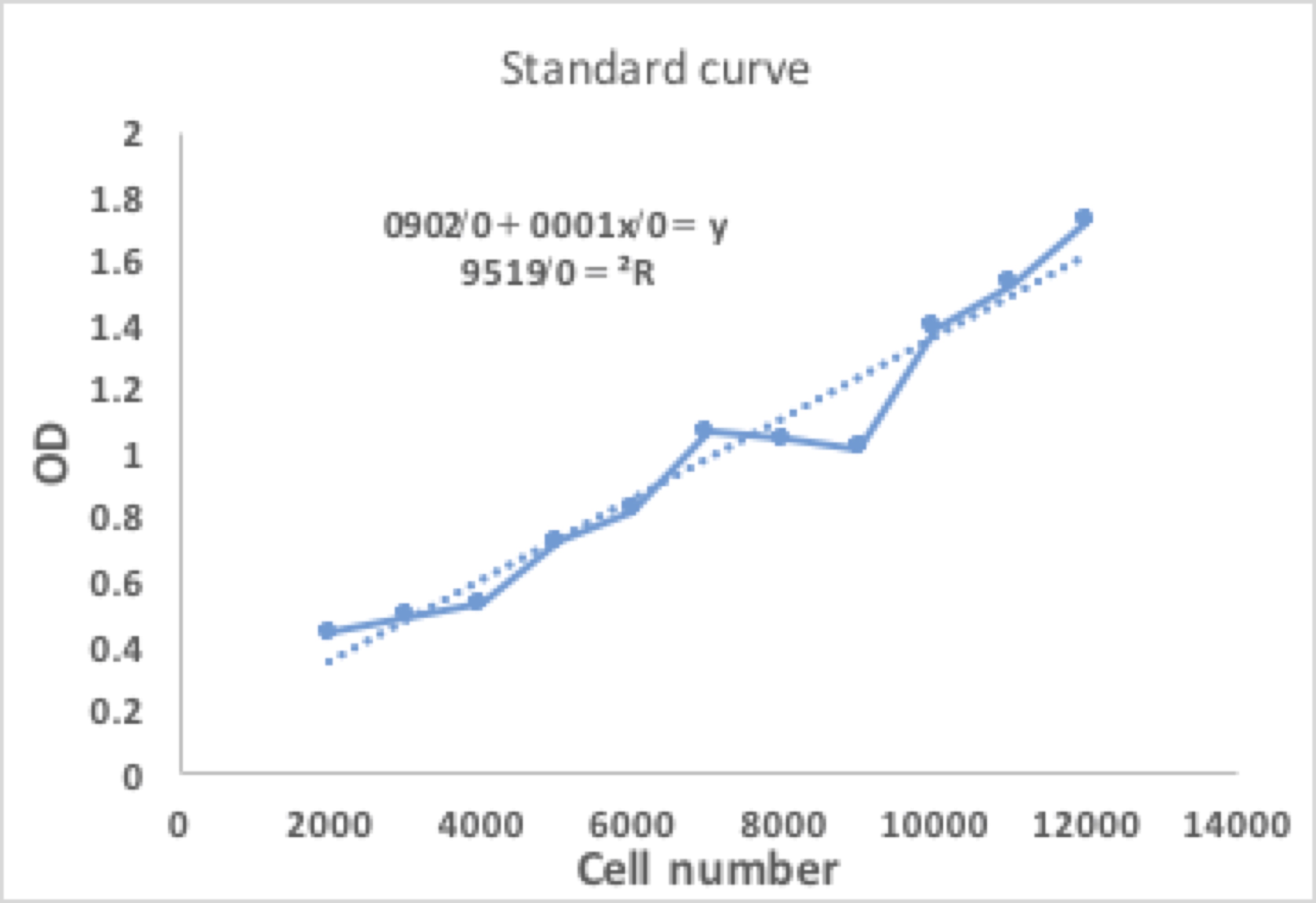

Supplement: S4 Fig — To optimize the number of MEFs for seeding in 96-well plate, MTT standard curve was performed. MEFs were seeded in 96-well plates at 1000 number intervals from 2000 to 12,000 (4 replicates). After 72 hours, MTT assay test performed without receiving any treatment. The results indicated a linear relationship between the number of cells and the intensity of optical density (OD). Since the aim of this study was to investigate the growth and proliferation of cells under appropriate treatments, half the maximum optical density suitable for MTT assay (OD = 1), can be used for this experiment. Therefore, out of 11 cell groups tested, a cell population of 4000 cells with average absorbance of 0.4–0.6 was selected for further studies. (JPG) [file pone.0247098.s005.jpg]
