# Supplementary material for: Combination treatment of dendrosomal nanocurcumin and low-level laser therapy develops proliferation and migration of mouse embryonic fibroblasts and alter TGF-β, VEGF, TNF-α and IL-6 expressions involved in wound healing process
Source: PLoS One. 2021 May 6;16(5):e0247098. doi: 10.1371/journal.pone.0247098 (PMC8101758; doi:10.1371/journal.pone.0247098)
Supplement: S1 Table — (PDF) [file pone.0247098.s006.pdf]

S1 Table. Primer sequences used to analyze the gene expression.

Gene	Primer sequences	Length（bp）
GAPDH	F5’-CCTGGAGAAACCTGCCAAGTA-3<br>R5’-GGCATCGAAGGTGGAAGAGT-3’	148 bp
TGF-b	F5-CGCAACAACGCCATCTATGAG-3’<br>R5’-CACATGTTGCTCCACACTTGA-3’	138 bp
VEGF-A	F5’-GGAGATCCTTCGAGGAGCACTT-3’<br>R5’-GGCGATTTAGCAGCAGATATAAGAA-3’	178 bp
TNF-α	F5’-CAGACCCTCACACTCACAAAC-3’<br>R5’-CAGCCTTGTCCCTTGAAGAGA-3’	170 bp
IL-6	F5’-GCAAGAGACTTCCATCCAGTTG-3’<br>R5’-ATAGACAGGTCTGTTGGGAGT-3’	123 bp
