# Supplementary material for: Combination treatment of dendrosomal nanocurcumin and low-level laser therapy develops proliferation and migration of mouse embryonic fibroblasts and alter TGF-β, VEGF, TNF-α and IL-6 expressions involved in wound healing process
Source: PLoS One. 2021 May 6;16(5):e0247098. doi: 10.1371/journal.pone.0247098 (PMC8101758; doi:10.1371/journal.pone.0247098)
Supplement: S3 Raw data — (PDF) [file pone.0247098.s009.pdf]

Raw data figure1. Effects of DNC on MEFs proliferation.

MTT assay. DNC. OD Read from Plate Reader.

Cntrl	0.25	0.5	0.75	1	2	4	6	8	10
0/799	0/901	1/102	1/529	0/857	0/98	0/819	0/64	0/772	0/504
0/789	0/97	1/315	1/042	1/084	0/812	1/082	0/558	0/857	0/569
0/722	0/959	1/164	1/144	0/933	0/923	1/131	0/584	0/841	0/584

MTT assay. DNC. The calculated percentage of cells relative to the control

Control	0.25	0.5	0.75	1	2	4	6	8	10
100	117/01	143/11	198/57	111/29	127/27	106/36	83/11	100/25	65/45
100	125/97	170/77	135/32	140/77	105/45	140/51	72/46	111/29	73/89
100	124/54	151/16	148/57	121/16	119/87	146/88	75/84	109/22	75/84

Raw data figure1. Effects LLLT on MEFs proliferation.

MTT assay. LLLT. OD Read from Plate Reader.

control	1j (0.31 j/cm2)	2j (0.63 j/cm2)	3j (0.95 j/cm2)	4j (1.27 j/cm2)	5j (1.59 j/cm2)	6j (1.91 j/cm2)	8j (2.54 j/cm2)	16j (5.09 j/cm2)	24j (7.64 j/cm2)	32j (10.19 j/cm2)	48j (15.28 j/cm2)	79.9j (25.47 j/cm2)
0/879	0/85	1/544	1/851	1/407	1/087	1/102	0/731	0/652	0/602	0/556	0/611	0/756
0/867	0/891	1/652	1/802	0/822	0/985	1/05	0/737	0/688	0/588	0/622	0/573	0/707
0/761	0/872	1/594	1/721	0/953	0/845	0/984	0/802	0/794	0/634	0/601	0/581	0/671

MTT assay. DNC. The calculated percentage of cells relative to the control

control	0.31 j/cm2	0.63 j/cm2	3j (0.95 j/cm2)	4j (1.27 j/cm2)	5j (1.59 j/cm2)	6j (1.91 j/cm2)	8j (2.54 j/cm2)	16j (5.09 j/cm2)	24j (7.64 j/cm2)	32j (10.19 j/cm2)	48j (15.28 j/cm2)	79.9j (25.47 j/cm2)
100	101/79	184/91	221/67	168/5	180/47	131/97	87/54	78/08	72/09	66/58	73/17	90/53
100	106/7	197/84	215/8	98/44	117/96	125/74	88/26	82/39	70/41	74/49	68/62	84/67
100	104/43	190/89	206/1	114/13	108/74	113/53	96/04	95/08	75/92	71/97	69/58	80/35

Raw data figure1. Effects combination treatment on MEFs proliferation.

MTT assay. combination treatment. OD Read from Plate Reader.

Control	0.5 µM DNC	0.75 µM DNC	0.63 j/cm2	0.95 j/cm2	0.5µM+0.63 j/cm2 (Simultaneous)	0.75µM+0.63 j/cm2 (Simultaneous)	0.5µM+0.95 j/cm2 (Simultaneous)	0.75µM+0.95 j/cm2 (Simultaneous)
0/484	0/791	0/767	0/692	0/867	0/948	0/772	1/121	1/29
0/431	0/779	0/742	0/725	0/793	0/883	0/94	1/064	1/113
0/325	0/683	0/897	0/778	0/888	0/925	0/932	1/003	1/726

MTT assay. combination treatment. The calculated percentage of cells relative to the control.

Control	0.5 µM DNC	0.75 µM DNC	0.63 j/cm2	0.95 j/cm2	0.5µM+0.63 j/cm2 (Simultaneous)	0.75µM+0.63 j/cm2 (Simultaneous)	0.5µM+0.95 j/cm2 (Simultaneous)	0.75µM+0.95 j/cm2 (Simultaneous)
100	191/52	185/71	167/55	217/76	229/53	227/6	271/42	417/9[illegible]
100	188/61	180/87	175/54	209/92	223/97	225/66	257/62	312/3[illegible]
100	165/37	217/19	188/37	192	213/8	186/92	242/85	269/4[illegible]
