# Supplementary material for: Combination treatment of dendrosomal nanocurcumin and low-level laser therapy develops proliferation and migration of mouse embryonic fibroblasts and alter TGF-β, VEGF, TNF-α and IL-6 expressions involved in wound healing process
Source: PLoS One. 2021 May 6;16(5):e0247098. doi: 10.1371/journal.pone.0247098 (PMC8101758; doi:10.1371/journal.pone.0247098)
Supplement: Attachment — Submitted filename: Dear reviewer2.docx [file pone.0247098.s010.docx]

Dear reviewer2

First, we would like to thank you for reviewing our manuscript. We also thank the reviewers for their careful reading and constructive suggestions. Reviewed manuscript was studied and revised according to reviewers’ comments. The comments were responded point by point and highlighted through the body of manuscript (in this file). It is our belief that the manuscript was substantially improved after applying the valuable reviewers’ comments

Reviewer #2: This is an interesting study that looks at the proliferation, motility of embryonic fibroblasts and the expression profiles TGF-β,VEGF , TNF-α and IL-6 following application of nanocurcumin and 450 nm light.

Author:

We appreciate the very positive and constructive comments from the reviewer. According to your comment, text was corrected and highlighted.

Before publication, please consider the following comments.
P 4 line 5: what culture medium was used?

Author:

The DMEM medium was used. The comment considered, I added and highlighted.

P4 4. Line 6: Although citations were included, some brief details of this optimized protocol should be included.

Author:

In Brief, different weight/weight ratios of dendrosome/curcumin were analyzed leading to the establishment of an appropriate ratio of 25:1. The loading of curcumin onto DNC was performed in which curcumin and dendrosome were co-dissolved in 5 mL of acetone followed by adding 5 mL of PBS, while stirring constantly. Acetone was evaporated by a rotary evaporator. The curcumin/dendrosome micelle solution was sterilized using a 0.22 μ syringe filter (Millex- LG, Millipore Co., USA). At the end, the prepared DNC was stored at 4 C in a light protected condition until use.

P4 Line 8: please add the exact model used and/or emission spectrum

Author:

The light source was a diode laser device provided by the RAADMED company from Iran at the wavelength of 450 nm with the output power of 75mW. The radiation spectrum of the diode source (SHARP GH04580A2G) is in the range of 440-460 nm (centred at 450 nm). The distance of sample from the light source was ﬁxed at 6cm with the beam area of light as 6cm^2^. **MEFs** were irradiated one time after passing 24 hours from seeding in a 96-well culture plate (the diameter of 7mm) for MTT assay) and within a petri dish with a diameter of 35mm for the rest of experiments. The irradiation was performed in a dark room. The irradiation time was automatically adjusted by the device just by setting the energy (Joule) of the radiation due to applying a constant power by the laser (Energy= Power x Time). Therefore, the cells were irradiated for 224 seconds for getting the energy of 17.9 Joule (with energy density of 0.63 J/cm^2^) and 337 seconds for the dose of 26.9 Joule (energy density of 0.95 J/cm^2^). For other doses, the time was set in the same way.

P4 line 34: please defined DMEM when first used.

Author:

The comment considered, I added and highlighted.
P5. Line 7: paint? Do you mean the pigment?

Author:

We changed this word to color.

The intensity of the **color** produced after dissolving the deposition of formazan in organic solvents such as dimethyl sulfoxide is measured spectrophoto-metrically.

P5 Line 10: indeed, the MTT assay is a measure of metabolic activity. Metabolism is highly dynamic so I am unsure one could claim that it is strictly associated with viability?

Author:

Thank you for your informative comment. We added (The reduction of MTT can only occur in metabolically active cells, so this assay represents the level of activity the cells) in the MTT section in the text. And we did cell cycle assay so we added (Since the aforementioned results were in line with the cell cycle assay results, the outcome was considered as the proliferation-inducing effect.) for proliferation.

P5 Line 12: what were the doses of nanocurcumin and/or light?

Author:

nanocurcumin (0.25-10 µM) and laser (0.31-25.47 j/cm^2^) alone and in combination.

P5. Line 20: which experimental groups? please be specific.

Author:

DNC alone (0.5, 0.75 μM), LLLT 450 nm alone (0.07, 0.10, 0.63 and 0.95 j/cm2 ) and the combination group (DNC (0.5, 0.75 μM) + LLLT 450nm (0.95 j/cm^2^)
P6. Line 4: were the proteins not extracted? Or was it just the supernatant? I would think it would be difficult to get enough protein concentration this way.

Author:

We used supernatant. We did Bradford assay for protein concentration. I added the result below. The Protein concentration was enough.

P6 Line 18: the authors need to provide the exact concentrations/doses used.

Author:

The comment considered, I added and highlighted.
P 7: Line 13. Were the experiments performed on 3 different days?

Author:

The experiments were performed on 3 different days.
